# The Impact of *DNMT3A/FLT3-ITD/NPM1* on Patients with Acute Myeloid Leukemia after Allogeneic Hematopoietic Stem Cell Transplantation

**DOI:** 10.4274/tjh.galenos.2018.2018.0274

**Published:** 2019-02-07

**Authors:** Long Su

**Affiliations:** 1 Jilin University First Hospital, Clinic of Hematology, Changchun, China

**Keywords:** Acute myeloid leukemia, Genetic mutations, DNMT3A, FLT3-ITD, NPM1

## To the Editor,

Recently, Ardestani et al. [1] published their excellent findings in this journal. They found that *DNMT3A* mutations alone do not affect the clinical outcomes of acute myeloid leukemia (AML) patients undergoing allogeneic hematopoietic stem cell transplantation (allo-HSCT), but when accompanied by *FLT3-ITD* mutations, the overall survival (OS) was significantly reduced and the relapse rate increased. *NPM1* mutations had no impact on either relapse-free survival or OS, but there was a significant difference between AML patients with and without *NPM1* mutations for relapse [1].

Integrative genomic analysis of de novo AML identified a subset of AML patients in which *DNMT3A*, FLT3, and *NPM1* mutations coexisted at a higher frequency than would be expected from chance occurrence [2]. Our unpublished data also showed that a close association could be observed among *DNMT3A*, *FLT3*, and *NPM1* mutations in patients with AML by factor analysis (p<0.05) based on 357 de novo AML patients analyzed by next-generation sequencing. A previous study demonstrated that younger (<60 years) patients with *DNMT3A/FLT3/NPM1* mutations had significantly shorter event-free survival (p=0.047) and a tendency towards shorter OS (p=0.095) compared to those in other mutation groups [3]. The adverse impact of *DNMT3A* mutations is more pronounced than that of *FLT3-ITD* among patients with *NPM1* mutations [3]. Accordingly, how did *DNMT3A/FLT3-ITD/NPM1* triple mutations influence the prognoses of AML patients who underwent allo-HSCT in this study? What about the impact of *DNMT3A* or *FLT3-ITD* on NPM1-mutated AML patients? Recent studies reported that variant allele frequencies of the *NPM1* and *FLT3-ITD* genes were closely related to long-term outcomes in patients with AML [4,5]. I wonder if there is information available on variant allele frequency in this subset of patients in order to re-analyze the impact of *NPM1* and *FLT3-ITD* on the prognoses of patients following allo-HSCT.

## Figures and Tables

**Figure 1:**
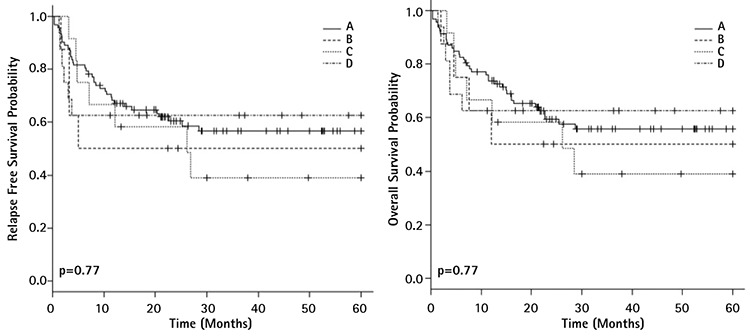
Survival curves of acute myeloid leukemia patients according to mutational status of *DNMT3A-/NPM1*: relapse free survival (left) and overall survival (right) (A=*DNMT3A-/NPM1*-, B=*DNMT3A+/NPM1*+, C=*DNMT3A+/NPM1*-, D=*DNMT3A-/NPM1*-).

**Figure 2:**
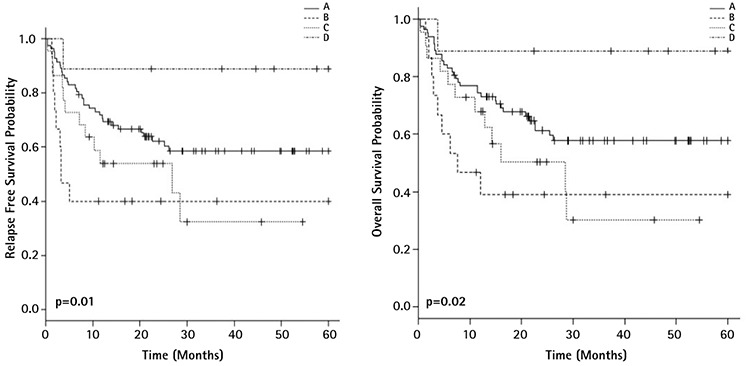
Survival curves of acute myeloid leukemia patients according to mutational status of *FLT3-ITD/NPM1*: relapse free survival (left) and overall survival (right) (A=*FLT3-ITD-/NPM1*-, B=*FLT3-ITD+/NPM1*+, C=*FLT3-ITD+/NPM1*-, D=*FLT3-ITD-/NPM1*+).

**Figure 3:**
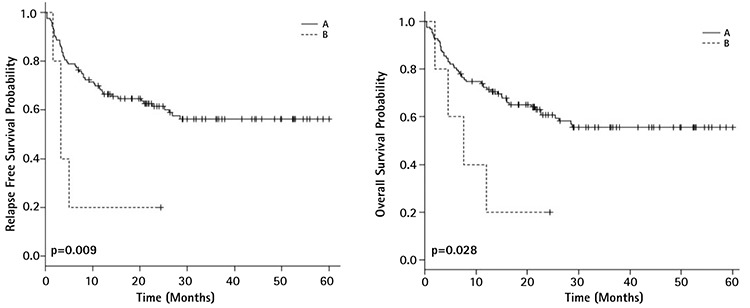
Survival curves of acute myeloid leukemia patients according to *DNMT3A/FLT3-ITD/NPM1* triple mutations: relapse free survival (left) and overall survival (right) (A=*DNMT3A/FLT3-ITD/NPM1*, B=others).
