# Supplementary material for: The Impact of DNMT3A/FLT3-ITD/NPM1 on Patients with Acute Myeloid Leukemia after Allogeneic Hematopoietic Stem Cell Transplantation
Source: Turk J Haematol. 2019 Feb 7;36(1):64–6. doi: 10.4274/tjh.galenos.2018.2018.0274 (PMC6373500; doi:10.4274/tjh.galenos.2018.2018.0274)
Supplement: Related texts

# Reply to the Authors

**Authors:** Bahram Chahardouli, Saeed Mohammadi, Mohsen Nikbakht, Shahrbano Rostami

## To the Editor,

We appreciate Dr. Long Su for his interest in our study and his useful comments about our article published in Turkish Journal Hematology, entitled “The impact of *DNMT3A/FLT3-ITD/NPM1* on patients with acute myeloid leukemia after allogeneic hematopoietic stem cell transplantation” [1]. Our study demonstrated that *DNMT3A* R882 mutations are not related to inferior survival in AML patients after allogeneic HSCT. Regarding his comments, we analyzed impact of *DNMT3A* and *FLT3-ITD* on *NPM1* mutated AML patients. Our finding indicated that considering *NPM1* and *DNMT3A* mutations together, no significant difference in OS and DFS was revealed (Figure 1).

*FLT3-ITD* mutation had a negative impact on both RFS and OS of patients with mutated *NPM1*. DFS and OS were significantly more favorable in patients with *FLT3-ITD-/NPM1+* compared to patients with other mutation subgroups (Figure 2).

Also, patients with *DNMT3A/FLT3-ITD/NPM1* triple mutations demonstrated a significantly worse DFS (p=0.009) and OS (p=0.028) (Figure 3) compared to all other patients (Figure 3). 

Unfortunately, in this study, information about the allele frequency of mutations was not available for most of patients, but in an ongoing prospective study, we are studying the effect of the allelic frequency of *FLT3-ITD* and NPM mutations on the clinical outcome of AML patients after HSCT.

Although there is more agreement on the poor prognostic role of DNMT3A mutations in AML patients [2], few studies have been conducted on the effect of these mutations on the clinical outcome of patients after allogeneic transplantation and there are disagreements over the results of these limited studies [3,4].

These differences can be due to coexistence of variable cytogenetic or molecular genetic aberrations in leukemia cells that could be effective in the clinical outcome of the patients. In the present study, only three genetic aberration were tested and additional genetic abnormality might impact the outcome of the patients. 

Taken together, our findings indicated that *FLT3-ITD* may be a more powerful adverse prognostic biomarker than *NPM1* and *DNMT3A* in AML patients after HSCT. Although the comments have allowed us to add useful information to our previous paper, our conclusion remains the same as in our published work.

**Best Regards,**

## References

[1] Ardestani MT, Kazemi A, Chahardouli B, Mohammadi S, Nikbakht M, Rostami S, Jalili M, Vaezi M, Alimoghaddam K, Ghavamzadeh A (2018). FLT3-ITD compared with DNMT3A R882 mutation is a more powerful independent inferior prognostic factor in adult acute myeloid leukemia patients after allogeneic hematopoietic stem cell transplantation: a retrospective cohort study. Turk J Hematol.

[2] Patkar N, Kodgule R, Kakirde C, Raval G, Bhanshe P, Joshi S, Chaudhary S, Badrinath Y, Ghoghale S, Kadechkar S, Khizer SH, Kannan S, Shetty D, Gokarn A, Punatkar S, Jain H, Bagal B, Menon H, Sengar M, Khattry N, Tembhare P, Subramanian P, Gujral S (2018). Clinical impact of measurable residual disease monitoring by ultradeep next generation sequencing in NPM1 mutated acute myeloid leukemia. Oncotarget.

[3] Wang H, Chu TT, Han SY, Qi JQ, Tang YQ, Qiu HY, Fu CC, Tang XW, Ruan CG, Wu DP, Han Y (2018.). FLT3-ITD and CEBPA mutations predict prognosis in acute myelogenous leukemia irrespective of hematopoietic stem cell transplantation. Biol Blood Marrow Transplant.

[4] Yang X, Shi J, Zhang X, Zhang G, Zhang J, Yang S, Wang J, Ke X, Fu L (2018). Biological and clinical influences of NPM1 in acute myeloid leukemia patients with DNMT3A mutations. Cancer Manag Res.
